# Successful bailout procedure for acute popliteal artery occlusion associated with EXOSEAL® vascular closure device: a case report

**DOI:** 10.1186/s13256-018-1950-2

**Published:** 2019-03-21

**Authors:** Ryota Urata, Tetsuya Nomura, Yusuke Hori, Kenichi Yoshioka, Hiroshi Kubota, Daisuke Miyawaki, Takeshi Sugimoto, Masakazu Kikai, Natsuya Keira, Tetsuya Tatsumi

**Affiliations:** Department of Cardiovascular Medicine, Kyoto Chubu Medical Center, 25, Yagi-Ueno, Yagi-cho, Nantan, Japan

**Keywords:** Case report, Complication, EXOSEAL, Vascular closure device, Acute limb ischemia

## Abstract

**Background:**

Vascular closure devices have been widely used to achieve rapid hemostasis after percutaneous catheterization procedures via the common femoral artery. The EXOSEAL vascular closure device is a device that can deliver a bioabsorbable polyglycolic acid plug to fill the subcutaneous puncture route at the groin for rapid hemostasis, and this device has a lower risk of arterial occlusion than other vascular closure devices.

**Case presentation:**

An 83-year-old Japanese man underwent percutaneous coronary intervention for a proximal stenosis in his left circumflex artery through a 7-Fr sheath from his right common femoral artery. We encountered acute popliteal artery occlusion associated with EXOSEAL vascular closure device. We detected the plug material of this device at the occluded lesion by intravascular ultrasound, and performed successful bailout stenting after pulling the embolus with an inflated balloon catheter up to the superficial femoral artery from the popliteal artery.

**Conclusion:**

Acute limb ischemia caused by an EXOSEAL vascular closure device is a very rare complication. Balloon angioplasty and stenting are considered to be effective options to deal with the plug dislodgement of an EXOSEAL vascular closure device. We must be prepared for every rare complication during endovascular treatment.

## Background

In the contemporary era of percutaneous coronary intervention (PCI), the use of vascular closure devices (VCDs) has become the standard method for achieving rapid hemostasis at the puncture site of the common femoral artery (CFA). Among them, the EXOSEAL (Cordis, NJ, USA) VCD can deliver a bioabsorbable polyglycolic acid plug to fill the subcutaneous puncture route at the groin. The EXOSEAL VCD is more advantageous than other VCDs because it leaves nothing behind inside the vessel, which reduces the risks of anchor-related luminal narrowing, occlusion, and distal embolisms. However, we encountered a case of acute popliteal artery (POP-A) occlusion associated with EXOSEAL VCD. We performed successful bailout stenting and confirmed that the postoperative course was uneventful in an 18-month follow-up study. We also reviewed the literature to discuss this rare complication caused by EXOSEAL VCD.

## Case presentation

An 83-year-old Japanese man underwent PCI for a proximal stenosis in his left circumflex artery through a 7-Fr sheath from his right CFA. We used an EXOSEAL VCD for hemostasis after we confirmed no calcification at the puncture site of the CFA. We performed the plug implantation according to the manufacturer’s instructions without any complications. However, we could not achieve complete hemostasis just with this procedure. Therefore, we added manual compression for 10 minutes in total, and we finally completed hemostasis. The next day, he complained of short distance intermittent claudication.

His past medical history was significant for hypertension, chronic kidney disease, paroxysmal atrial fibrillation, and silent myocardial ischemia. His regular medications were dual-antiplatelet therapy of aspirin (100 mg) + prasugrel (3.75 mg), and an oral factor Xa inhibitor (apixaban, 2.5 mg twice daily). There was no family history. He was a farmer. He did not smoke tobacco and he was a social drinker. His physical examination revealed an absence of a right popliteal pulse. His right lower extremity was pallid and perishing cold without ulceration. There was no motor and sensory loss. His blood pressure was 170/75 mmHg, pulse rate was 70 beats/minute, oxygen saturation was 98%, and body temperature was 36.5 °C. The laboratory examination findings were as follows: serum creatinine 1.28 mg/dL, creatine phosphokinase (CPK) 1236 U/L, aspartate aminotransferase (AST) 45 U/L, alanine aminotransferase (ALT) 25 U/L, lactate dehydrogenase (LDH) 229 U/L, C-reactive protein 0.7 mg/dL, white blood cell count 4.63 × 103/μL, red blood cell count 11.6 × 106/μL, and platelet count 176 × 103/μL. His blood culture was negative.

A chest X-ray demonstrated no abnormal findings. Electrocardiography showed normal sinus rhythm and complete left bundle branch block. On echocardiography, the left ventricular ejection fraction was 42% and diffuse motion abnormality in his left ventricle was observed. His ankle-brachial pressure index could not be measured on his right leg. Doppler ultrasound demonstrated no stenosis or occlusion in the visible area of his right CFA and superficial femoral artery (SFA). However, in his right POP-A, the acceleration time was prolonged up to 125 msec, and the blood flow pattern was monophasic. Therefore, severe stenosis or occlusion in the distal SFA was suspected, and we performed an emergency angiography.

**Fig. 1:**
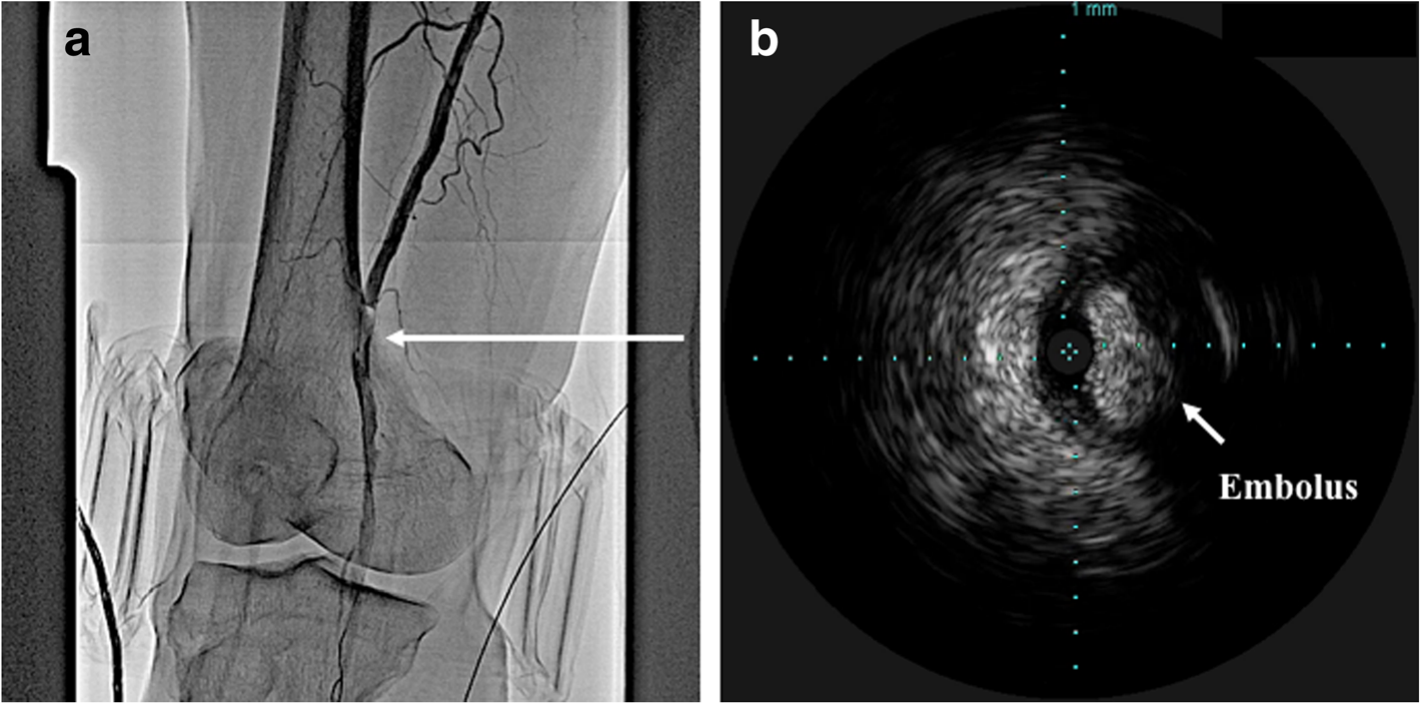
**a** The angiography showed subtotal occlusion (arrow) at the proximal popliteal artery. **b** Intravascular ultrasound image demonstrated a smooth-surfaced high-density homogenous structure (arrow) which was suspected as a polyglycolic acid fiber plug material of the EXOSEAL vascular closure device

**Fig. 2:**
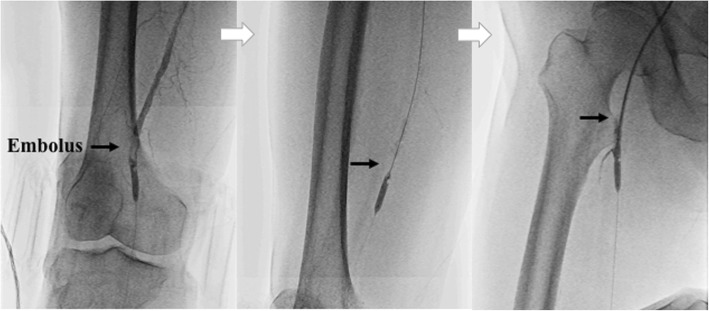
We pulled the embolus (arrows) up to the proximal superficial femoral artery, similar to using a Fogarty balloon catheter, and compressed it on the arterial wall by 5.0 × 20-mm balloon catheter inflation

**Fig. 3:**
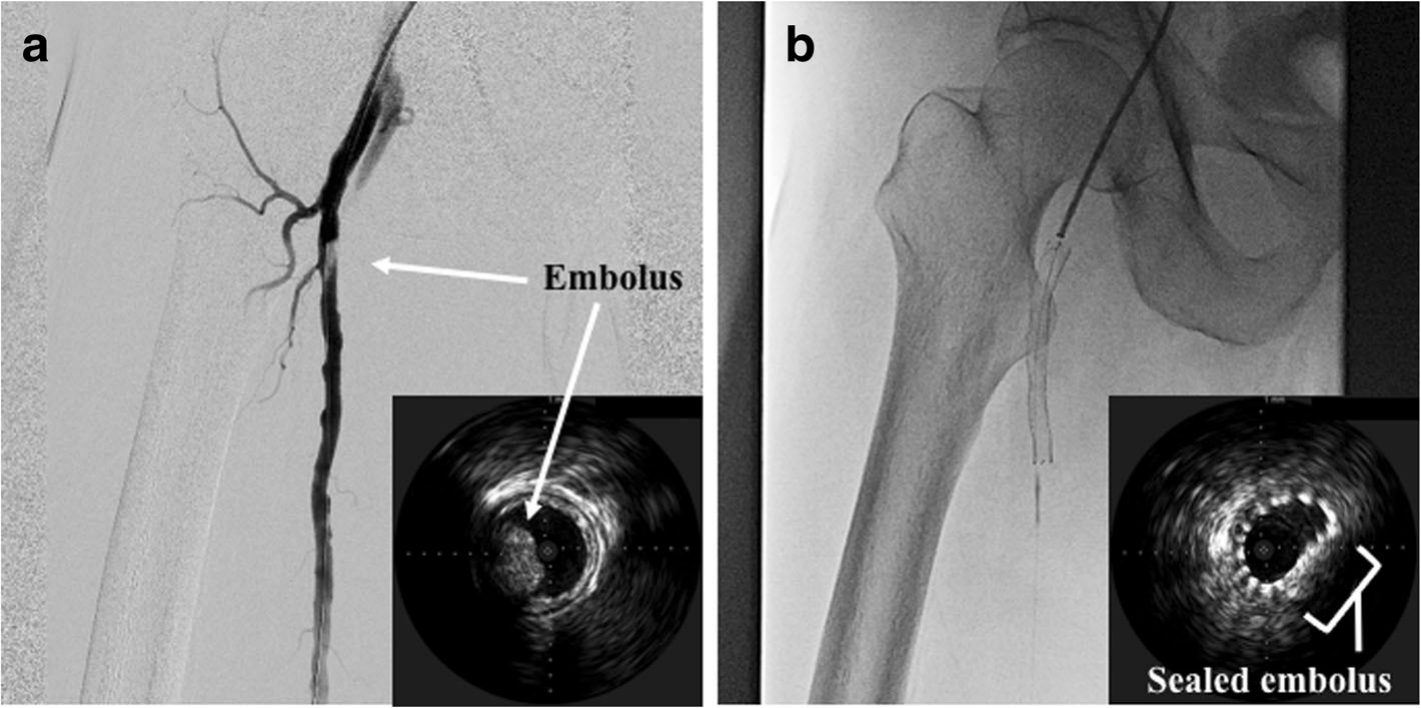
**a** We confirmed the embolus (arrows) was attached to the proximal superficial femoral artery by angiography and intravascular ultrasound. **b** We implanted a 7.0 × 60-mm self-expandable nitinol stent to seal the embolus (square bracket)

**Fig. 4:**
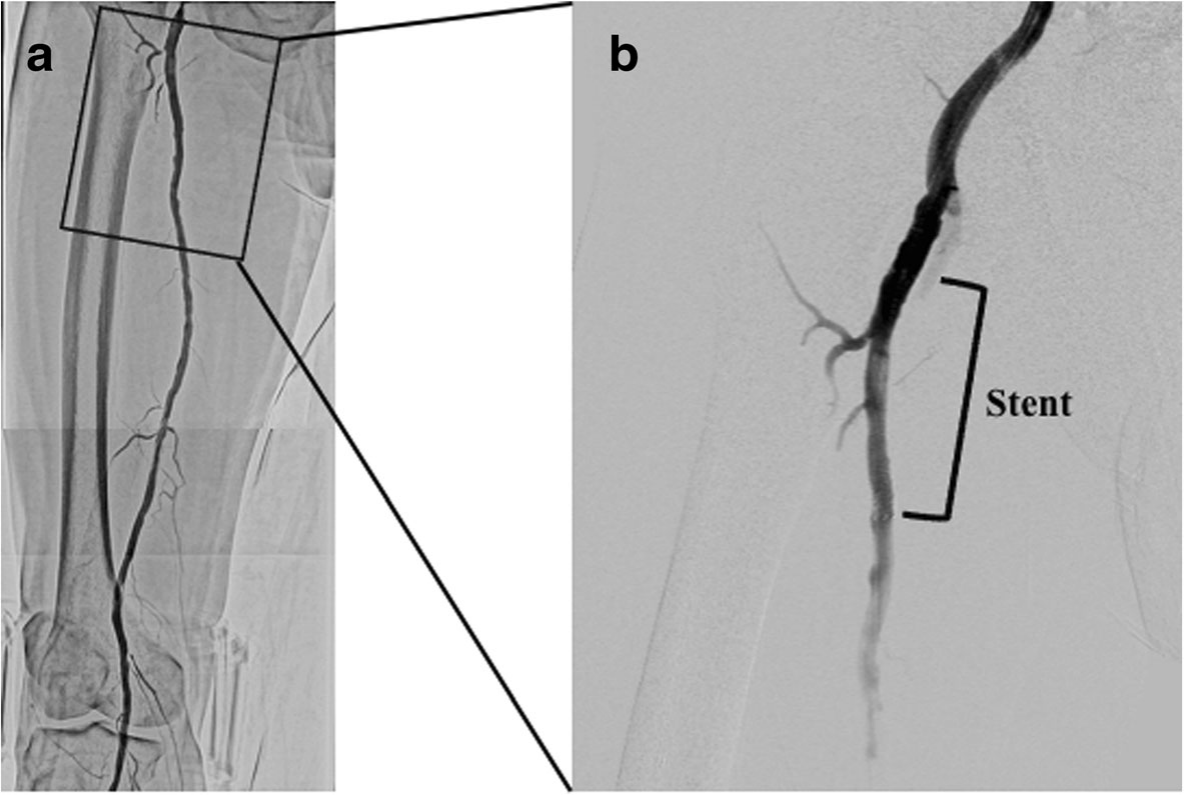
**a** Final angiography demonstrated a favorable blood flow in the right lower extremity. **b** There was no radiolucent mass in the proximal superficial femoral artery where the embolus was sealed with a self-expandable stent

The angiography showed no significant stenosis from the right common iliac artery to the CFA. However, a subtotal occlusion at the proximal site of POP-A was observed, and we moved on to endovascular treatment (EVT) using a 6-Fr guiding sheath via his left CFA. First, we pressed his POP-A by a cuff at 200 mmHg for the purpose of avoiding a distal embolization due to further treatment. We performed manual aspiration using a 6-Fr guiding catheter, but no embolus was aspirated and we could not recanalize the artery. Next, we passed a 0.014-inch guidewire with the support of intravascular ultrasound (IVUS). IVUS imaging demonstrated a smooth-surfaced high-density homogenous structure that was suspected to be polyglycolic acid fiber plug material of the EXOSEAL VCD (Fig. 1). We tried embolectomy by pulling an ordinary 5.0 × 20-mm inflated balloon catheter back from the POP-A into the 6-Fr guiding catheter in the SFA, similar to using a Fogarty balloon catheter. However, the embolus was too large to be collected into the 6-Fr guiding catheter. Therefore, we decided to seal the material on the arterial wall with a stent. To avoid stenting the POP-A, we pulled the embolus up to the proximal SFA and compressed it on the arterial wall by 5.0 × 20-mm balloon catheter inflation for 30 seconds (Fig. 2). We confirmed that the embolus was attached to the arterial wall of the proximal SFA by angiography and IVUS. Then, we deployed a 7.0 × 60-mm self-expandable nitinol SMART (Cordis, CA, USA) stent to seal the embolus (Fig. 3). Final angiography demonstrated a favorable blood flow in our patient’s right lower extremity (Fig. 4). After the procedure, the value of his ankle-brachial index (ABI) was normalized and his symptoms completely disappeared. Angiography conducted 11 months postoperatively demonstrated no significant restenosis in the stent of his right CFA. Doppler ultrasound performed 18 months postoperatively showed no stenosis or occlusion in his right CFA and SFA. His postoperative course was uneventful in an 18-month follow-up study.

## Discussion and conclusions

We encountered a case of acute POP-A occlusion associated with EXOSEAL VCD, and performed successful bailout stenting after pulling the embolus with an inflated balloon catheter up to the SFA from the POP-A. Acute limb ischemia (ALI) caused by EXOSEAL VCD is a very rare complication. In addition, no effective bailout procedure has been established. To the best of our knowledge, this is the first case of successful bailout EVT for such a complication.

The use of VCDs has now become part of the standard method for achieving rapid hemostasis at the puncture site of CFA. VCDs have considerable potential for reducing procedure time, length of hospital stay, and duration of restricted ambulation. To date, several VCDs employing different mechanisms for hemostasis have become available. The safety and efficacy of these VCDs have been confirmed for retrograde access usage in several studies [1]. However, they sometimes cause rare complications such as luminal stenosis, occlusion, or peripheral embolism. VCD-induced lower limb ischemia has been reported infrequently, and was only encountered in 0.3% of patients in a recent meta-analysis [2].

The EXOSEAL VCD adopts a completely absorbable polyglycolic acid plug. The absorbable plug is fully enclosed in the distal portion of the delivery shaft. The plug applicator positions and deploys the absorbable plug to the extravascular surface of the arterial access site through the existing sheath without the need for a sheath exchange before device deployment. The device has two safety mechanisms to prevent intravascular deployment of the plug. The first one is a capillary bleed backflow visual indicator. While the plug is still inside the vessel or within the vessel wall, the backflow of the blood can be observed. The second one is an indicator wire at the tip of the device. Only when the indicator wire is stretched by the vascular wall, can the deployment button be pushed. Unlike other VCDs, the EXOSEAL VCD does not leave any foreign bodies, such as an anchor, nitinol clip, or sutures, inside the vessel owing to these two safety mechanisms. Therefore, the EXOSEAL VCD exhibits relatively lower risks of anchor-related luminal narrowing, occlusion, and distal embolisms than other VCDs.

The 2009 ECLIPSE trial reported by Wong *et al.* remains the only available randomized trial that evaluated the EXOSEAL VCD system regarding patient safety and successful hemostasis [3]. In this study, no major complications were observed (0/267 cases). Kamusella *et al.* reported a case of device related stenosis (0.1%) among 1000 patients in whom the EXOSEAL VCD was used. The stenosis was detected by the partially intravascular position of the plug, but hemodynamically significant stenosis was not evident [4]. As described above, complications of arterial occlusion induced by EXOSEAL VCD are thought to be very rare. To the best of our knowledge, only three cases have been reported to date. Maxien *et al*. reported a case of ALI caused by an EXOSEAL VCD. They performed endovascular repair by balloon angioplasty and stenting [5]. Takasawa *et al*. reported two cases of femoral artery occlusion after using this device. Both cases underwent surgical repair [6].

The causes of acute artery occlusion associated with the EXOSEAL VCD are considered as follows. First, if an operator pushes the EXOSEAL VCD system itself toward the body while simultaneously pushing the deployment button, the plug may be released into the vascular lumen. Second, when the indicator wire at the tip of the EXOSEAL VCD gets stuck in a stent or vascular atherosclerotic calcifications before the indicator wire reaches the vascular wall, the visual indicator changes its color from white to black. In this case, although blood backflow is still observed, there is a risk of the operator pushing the deployment button based on the indicator marker. Third, if an additional manual compression at the puncture site after deployment of the EXOSEAL VCD is too strong, the plug may partially or wholly fall into the vessel.

In our case, we confirmed the absence of atherosclerotic lesions in the CFA. We pushed the deployment button after confirming the visual indicator while tightly fixing the EXOSEAL VCD system to not move at all. Through this procedure, we most likely did not leave the plug in the vessel at the time of deployment. However, we added relatively strong compression on the puncture site for 10 minutes in total because we could not achieve complete hemostasis just with initial deployment of the plug. This extra manual compression may have caused the plug to dislodge partially or wholly from the right position into the vascular lumen. We consider that manual compression after EXOSEAL VCD use should be as gentle as possible, but enough to complete hemostasis.

When ALI is induced by plug dislodgement of EXOSEAL VCD, EVT is considered an effective option. Aspirating the plug through a guiding catheter seems impossible because the plug becomes too large in the vessel to be collected into the guiding catheter. Grasping the embolus by biopsy forceps risks breaking the embolus into small pieces and distally scattering them. The plug swelling in the blood is relatively soft and cylindrical, thus, we consider the stenting method to seal the plug material to be an effective and minimally invasive resolution. It is desirable to use a self-expandable stent because the plug material is completely absorbed in the body by 90 days after implantation, by which time enough expansion of the stent is expected after the plug vanishes. When the plug is located in a non-stenting zone, like our case, we have to move the embolus to be sealed by a stent to the appropriate location. At the same time, it is important that we press the POP-A from the outside and block the blood flow to lower extremities to prevent distal embolization.

Here we demonstrated a successful bailout procedure for acute POP-A occlusion associated with EXOSEAL VCD. Although the rate of ALI caused by EXOSEAL VCD is not higher than other VCDs, we always have to prepare for every rare complication during EVT. Balloon angioplasty and stenting are considered to be effective options to deal with dislodging of the EXOSEAL VCD plug.

## Abbreviations

ALI: Acute limb ischemia
CFA: Common femoral artery
EVT: Endovascular treatment
IVUS: Intravascular ultrasound
PCI: Percutaneous coronary intervention
POP-A: Popliteal artery
SFA: Superficial femoral artery
VCD: vascular closure device

## References

[1] Krishnasamy VP, Hagar MJ, Scher DJ, Sanogo ML, Gabriel GE, Sarin SN. Vascular closure devices: Technical tips, complications, and management. Tech Vasc Interv Radiol. 2015;18(2):100–12. 10.1053/j.tvir.2015.04.008 26070622

[2] Biancari F, D’Andrea V, Marco CD, Savino G, Tiozzo V, Catania A. Meta-analysis of randomized trials on the efficacy of vascular closure devices after diagnostic angiography and angioplasty. Am Heart J. 2010;159(4):518–31. 10.1016/j.ahj.2009.12.027 20362708

[3] Wong SC, Bachinsky W, Cambier P, Stoler R, Aji J, Rogers JH, *et al.* A randomized comparison of a novel bioabsorbable vascular closure device versus manual compression in the achievement of hemostasis after percutaneous femoral procedures: the ECLIPSE (Ensure's Vascular Closure Device Speeds Hemostasis Trial). J Am Coll Cardiol. 2009;2(8):785–93. 10.1016/j.jcin.2009.06.006 19695549

[4] Kamusella P, Wissgott C, Jahnke T, Brossmann J, Scheer F, Andressen R, *et al.* Percutaneous Vascular Closure System Based on an Extravascular, Bioabsorbable Polyglycolic Plug (ExoSeal): Results from 1000 Patients. Clin Med Insights Cardiol. 2014;8(Suppl2):49–52. 10.4137/CMC.S15229 PMC4463796 26085789

[5] Maxien D, Behrends B, Eberhardt KM, Saam T, Thieme SF, Reiser MF, *et al.* Endovascular treatment of acute limb ischemia caused by an intravascularly deployed bioabsorbable plug of a vascular closure device. Vasa. 2013;42(2):144–8. 10.1024/0301-1526/a000260 23485843

[6] Takasawa Y, Mizuno S, Yamaguchi J, Suzuki M, Tsuchida M, Saga M, *et al.* Cases of Femoral Artery Occlusion after using the Exoseal Closure Device: Including Experiment in Vascular Model. Cardiovasc Interv Ther. 2015;7(3):81–5.

